# A case with Behçet’s disease involving erosive Metacarpophalangeal joint arthritis: the value of ultrasonography in the diagnosis of an Erosion

**DOI:** 10.1186/s12880-020-00461-8

**Published:** 2020-06-03

**Authors:** Cevriye MÜLKOĞLU, F. Figen AYHAN

**Affiliations:** 1 Department of Physical Medicine and Rehabilitation, Health Sciences University Ankara Training and Research Hospital, Altındağ/ANKARA, Ulucanlar Street, 06230 Ankara, Turkey; 2 Medicana International Hospital, Department of Physical Medicine and Rehabilitation, Ankara, Turkey

**Keywords:** Behçet’s disease, Destructive arthritis, Ultrasound

## Abstract

**Background:**

Behçet’s disease (BD) is a chronic multisystemic vasculitis that may emerge with musculoskeletal system involvements, oral-genital recurrent aphthae, mucocutaneous lesions, and ocular symptoms. Arthritis in BD is usually non-erosive and not related to crippling disease. Erosive arthropathy is a very rare manifestation of BD.

**Case presentation:**

Herein, we present a 60-year-old male patient suffering from BD for 33 years with erosive arthritis in his second metacarpophalangeal joint. After we assessed his finger by X-ray, we diagnosed erosive arthropathy quickly by musculoskeletal ultrasonography. In addition, a systematic literature search was performed via the PubMed and Scopus databases using the keywords, ‘Behçet’s disease [AND] erosive/destructive arthritis’.

**Conclusions:**

Erosive arthritis due to BD can be evaluated by ultrasonography in an easy, fast and cost-effective manner. The literature search between 1985 and December 2019 revealed a total of 19 patients with peripheral erosive arthropathy related to BD and the characteristics of the results are summarized in the paper.

## Background

Behçet’s disease (BD) is a vasculitic disorder with multisystem involvement, such as the central nervous, locomotor and gastrointestinal systems, and blood vessels. Symptoms include recurrent oral and genital ulcerations, hypopyon uveitis, cutaneous lesions, and peripheral arthritis [1]. Almost 40–70% of the cases with BD have rheumatologic complaints. BD often causes non-destructive and non-deforming self-limiting arthritis. Joint involvement in BD is usually recurrent, inflammatory, symmetric or asymmetric, and mono or oligoarthritis [1–5]. Erosive arthritis has been rarely reported in patients with BD [1, 4, 6–15]. Sometimes, BD can mimic rheumatoid arthritis with polyarticular involvement [8]. Rarely, secondary ankylosing spondylitis, axial involvement, or isolated sacroiliitis can occur in BD [16–18]. In this study, we evaluated patients with peripheral destructive arthritis but did not include those with sacroiliitis due to BD.

In this report, we present the erosive arthritis of the metacarpophalangeal (MCP) joint in a patient suffering from BD for 33 years. We performed a literature search focusing on English articles using the keywords, ‘Behçet’s disease and erosive/destructive arthritis’. A total of 10 articles were identified and carefully reviewed, and the reported erosive joint involvements were summarized.

## Case presentation

**Fig. 1:**
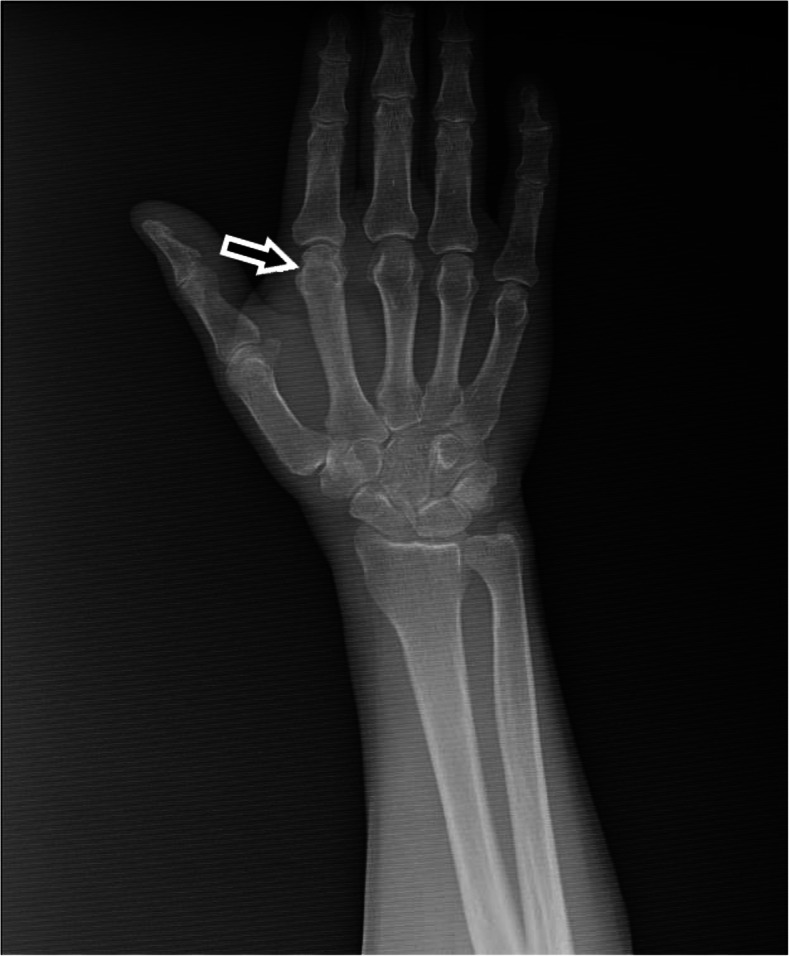
Conventional radiograph of the right hand and second MCP joint of our patient. The arrow shows the suspected erosion in the second MCP joint

**Fig. 2:**
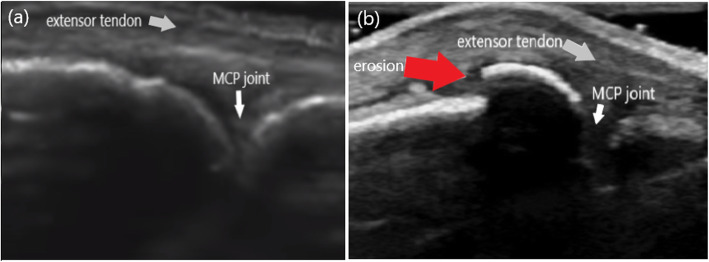
The longitudinal images of the second MCP joint in dorsal aspect (**a**): A normal articular surface (white arrow) of the second MCP joint. (**b**): The appearance of the erosion (red arrow) as the step-down contour defect

**Fig. 3:**
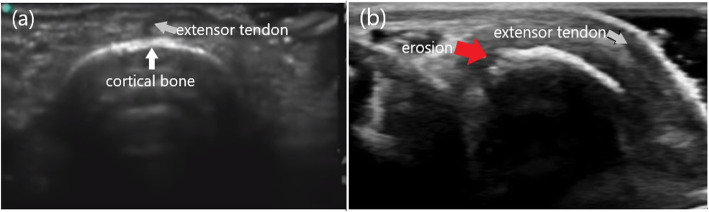
The transverse images of the second MCP joint in dorsal aspect (**a**): A normal cortical bone (white arrow) structure of the second MCP joint. (**b**): The appearance of an erosion (red arrow) in the bone

A 60-year-old male patient was presented to our outpatient clinic with the complaints of swelling and pain in the second finger of his right hand. BD had been diagnosed 33 years earlier. He had experienced swelling in his right knee about 30 years earlier, but he was symptom-free after joint aspiration. Then, 20 years ago, he had a swollen left ankle, which improved spontaneously within 10 days. At that time, he used colchicine for 2 years, but stopped this drug himself due to gastrointestinal adverse effects. At the time of presentation to our clinic, the patient had not used colchicine or other medication for the last 10 years. He mentioned that he had been having difficulty using his hand due to swelling and pain in the second finger for the last 3 months. On physical examination, there was pain and swelling in his second MCP joint. He had no oral or genital aphthae. The fundus examination revealed no uveitis. The erythrocyte sedimentation rate was 9 mm/h and C-reactive protein was 8.5 mg/liter (normal range is 0–5). Rheumatoid factor and antinuclear antibody were negative. HLA-B51 antigen was positive. The kidney and liver functions and other blood tests were within normal limits. Since we suspected destructive changes in the second MCP joint of the right hand on plain radiograph (Fig. 1), we evaluated the patient by GE Logiq 5 ultrasonography (US) in our department. The US examination was performed from dorsal aspect of the second MCP joint with longitudinal and transverse scans. A step-down contour defect of the erosion was observed in the second MCP joint on longitudinal image. The normal and erosive articular surface of the second MCP joint were indicated, on longitudinal images in Fig. 2 and on transverse images in Fig. 3. After the diagnosis of the bone erosion, the patient was prescribed a weekly dose of 10 mg methotrexate. At the three-month follow-up, his complaints had almost completely been resolved. Written informed consent was obtained from the patient for the publication of this report.

We searched PubMed, MEDLINE, EMBASE, Scopus, and Web of Science using the keywords, ‘Behçet’s disease [AND] erosive arthritis’ and ‘Behçet’s disease [AND] destructive arthritis’. The original articles, letters and case reports published in English between 1985 and December 2019 were identified. Publications containing abstracts with limited information, comments or papers written in languages other than English, irrelevant articles, and duplicates were excluded.

We carefully reviewed 10 articles and the references therein. Five articles were not further evaluated because their main text was in French. Eight of the 10 articles were case reports/letters and two were original articles/reviews. Most case reports were published after the year 2000. Table 1 presents the characteristics of the 19 patients with erosive arthropathy reported in the evaluated articles. The mean age of these patients was 43.89 (range 19–72) years, and 10 were female. The main presenting symptoms were usually monoarthritis or asymmetrical oligoarthritis.

**Table 1:** Previously published cases of erosive arthritis associated with BD

Authors	Year	Number of patients (n)	Gender	Age (years)	Localisation of erosive arthritis	References
**Jawad et al.**	1986	1	FM	31	Wrists and intercarpal joints.	[14]
**Armas et al.**	1992	1	M	52	Scaphoid, ulnar styloid process, MCP joints, MTP joints	[9]
**Momohara et al.**	2001	1	FM	52	bilateral fingers, wrists, and left ankle	[15]
**Düzgün et al.**	2003	1	FM	35	First MTP and first PIP joints of the right foot.	[4]
**Aydın et al.**	2005	1	M	53	Carpal bones at the right wrist, distal end of the right radius and ulna, MTP joints, right hallux IP joint.	[10]
**Sugawara et al.**	2009	3	FM	58 55 48	DIP, PIP, radiocarpal, ulnar stiloid process	[11]
**Frikha et al.**	2009	8	6 M, 2 FM	19–58	Knees, elbows,wrist, tarsal scaphoid, sterno-clavicular joint, feet	[13]
**Daoud et al.**	2011	1	M	36	Bilateral MTP and asymmetrical IP joint of feet	[1]
**Tuncay et al.**	2013	1	FM	72	Bilateral elbows,wrists, MCP and PIP joints	[8]
**Jung et al.**	2018	1	FM	48	feet	[12]
**Current case**	2020	1	M	60	Second MCP joint of the right hand.	

*M* Male; *F* Female; *MTP* Metatarsophalangeal; *MCP* Metacarpophalangeal joint; *PIP* Proximal interphalangeal joint; *DIP* Distal interphalangeal joint; *IP* Interphalangeal joint

## Discussion and Conclusions

In this paper, we report a case of BD with erosive arthritis, who had presented to our clinic with the complaints of swelling and pain in his MCP joint. The patient fulfilled the International Study Group (ISG) classification criteria for BD [19].

ISG classification criteria include recurrent oral aphthae (minor or major aphthous or herpetiform lesions recurring at least three times a year and detected by the physician), recurrent genital ulcers, eye lesions (anterior or posterior uveitis, retinal vasculitis, etc.), skin lesions (pseudofolliculitis or papulopustular lesions, acneiform nodules, etc.), and a positive pathergy test. BD diagnosis is made up with the presence of recurrent oral aphthae and together two of the remaining findings.

Although the large joints of the lower limb are commonly affected in BD, the occurrence of erosive arthritis in the small joints of the upper extremity is a rare manifestation.

BD is a multisystemic disease with an unknown etiology, mainly characterized by recurrent oral and genital ulcerations, and chronic relapsing uveitis. Vascular, neurological, musculoskeletal, and gastrointestinal systems can be involved. BD is most active during young adulthood, causing serious disability and significant impairment of quality of life.

Peripheral arthritis in the large joints of the lower limb and sacroiliitis are some of the musculoskeletal disorders that may be found in BD. The inclusion of BD among seronegative spondyloarthropathies and whether sacroiliitis develops in BD are still being debated [16]. Chamberlain et al. did not find any evidence of increased prevalence of sacroiliitis in BD [18]. There were no findings of sacroiliitis in our patient. Therefore, in the review part of this study, we evaluated patients with peripheral erosive arthritis due to BD, not sacroiliitis.

BD usually causes subacute, non-destructive and non-crippling arthritis. Erosive arthritis is an uncommon presentation of this disease. Among the reviewed publications, Frikha et al. retrospectively examined 553 patients with BD and found that eight had erosive arthritis, of whom six were female and two were male. The age range of these patients was 19 to 58 years. The main presenting symptom was monoarthritis or asymmetrical oligoarthritis. The authors used X-ray examinations to evaluate the erosive joints. The localizations of erosive arthritis were the knees, elbows, wrist, tarsal scaphoid, sternoclavicular joint, and feet [13]. We evaluated the MCP joint of our patient using US and confirmed that he had monoarthritis.

Sugawara et al. observed erosive arthritis in three of four patients with BD, all women. They used conventional radiography and magnetic resonance imaging (MRI) to elucidate the erosive pattern. One patient with the non-erosive synovitis of the wrist, one with wrist synovitis with a minimal erosion, and two with erosive arthritis of the distal interphalangeal joint [11].

Düzgün et al. showed erosive arthritis in the first metatarsophalangeal and first proximal interphalangeal joints of the right foot by calcaneal enthesopathy using X-rays [4]. In our report, the patient with an erosion without any enthesopathy or tendinopathy was detected by US. Sometimes, BD can mimic rheumatoid arthritis with a polyarticular presentation. Tuncay et al. reported a BD case with erosive arthritis in the small joints of hands, mimicking rheumatoid arthritis. They also evaluated the patient by direct radiography. Bilateral elbows, wrists, MCP, and proximal interphalangeal joints were found to be involved, and the findings were radiologically similar to those of rheumatoid arthritis [8].

The course of BD is favorable, patients have good response to colchicine, and arthritis attacks usually improve within two to 4 weeks without any joint damage. The joints affected frequently in BD are large joints, especially those of the knee, ankle, elbow, and wrist [3, 5]. The shoulder, hip, and small joints of the hands and feet are rarely affected. The occurrence of erosive arthritis in the MCP joint is a rare manifestation of BD. We found only two reports with erosive arthritis in the MCP joint related to BD [8, 9].

Radiological erosive changes detected in the joint are important in predicting the prognosis of arthritis. The early diagnosis of arthritis allows the early initiation of disease-modifying antirheumatic drugs, preventing bone erosion changes and reducing disease progression [20]. Radiographs have traditionally been the main diagnostic method for imaging patients with arthritis, revealing findings, such as soft tissue swelling, periarticular osteopenia, loss of joint space, joint subluxation, and marginal erosions. However, if synovial proliferation or joint effusion is small, soft tissue swelling and effusions are difficult to diagnose by radiography. Baillet et al. showed that US was more effective than conventional radiography in detecting bone erosions and had an efficacy comparable to MRI [21]. Similarly, Wakefield et al. compared the ability of US to detect MCP joint erosions in patients with rheumatoid arthritis using conventional radiography and reported that US was a reliable technique that detected more erosions than conventional radiology, especially in early rheumatoid arthritis [22].

There are some advantages and disadvantages to consider when deciding on whether to use US or MRI as the imaging modality. US allows the clinician to easily examine the contralateral side of the joint or other joints, as well as facilitating a clinical evaluation of the patient during imaging. In addition, US is easily available in many centers and less costly than MRI. However, the disadvantages associated with the US assessment of the joints include the inability to visualize the internal structure of the bone or assess bone edema, and the technique being operator-dependent. US can be time-consuming and has a long learning process for an inexperienced operator. MRI provides a more general view of the joint, including joint surfaces and the internal structure of the bone. The disadvantages of using MRI include motion artifacts and the invasive administration of contrast media. MRI also has relatively lower resolution compared to US, and multiple joints can be difficult to display as a contrast application is required to reliably differentiate synovium from effusion [23].

Nepal et al. conducted a study on the typical and atypical imaging features of hand tumors and discussed the advantages of radiography, US, and MRI under different conditions. Although plain radiography cannot show soft tissue tumor in most cases, it can sometimes provide important information about tumors. US is used less frequently, but it is a useful method for soft-tissue hand tumors. Through its high-resolution images, it can distinguish between cystic and solid tumors, as well as displaying the nerves entering into and exiting from nerve sheath tumors. Color Doppler US can provide important information about vascularity. MRI is often used to characterize soft-tissue tumors. MRI can also help the radiologist understand tumor histopathology [24].

On US, an erosion is visualized as the absence of intra-articular bone surfaces in two planes perpendicular to each other. Bone erosions are defined as bony lesions that have a juxtaarticular joint localization and typical images that can be seen in at least two planes, and are sharply limited by cortical refraction observed in at least one plane. US is more sensitive than conventional radiography in detecting joint damage [22]. It is possible to detect erosions by US in almost half of the patients with radiographs showing non-erosive findings.

US can also be used to visualize non-erosive arthritis. US can detect pre-erosion synovitis. Both US and MRI have been shown to be superior to clinical evaluation and radiography in detecting the presence of synovitis [23]. On US, synovial effusion and hypertrophy are indicated by abnormal hypoechoic or anechoic intra-articular tissues.

The use of musculoskeletal system US in the diagnosis and treatment of inflammatory arthritis is gradually increasing among rheumatologists worldwide, in the last decades. Dohn et al. reported that US and MRI showed MCP joint erosions in 17 patients with rheumatoid arthritis at 91 and 96% specificity, respectively. Their study strongly demonstrated that erosions detected by US and MRI in patients with rheumatoid arthritis represented true erosions [25]. In another study of the same author, the 2nd-5th MCP joints of 49 patients with rheumatoid arthritis were examined by both US and computed tomography. The authors reported that US had an overall sensitivity of 44%, specificity of 95% and accuracy of 78% for the detection of bone erosions in rheumatoid arthritis MCP joints, with computed tomography as the reference method. The sensitivity/specificity/accuracy of US increased to 71%/95%/90%, when only areas with good accessibility (radial 2nd MCP, ulnar 5th MCP and all dorsal/palmar aspects) were included. Although US detected 95% of erosions with bone volume loss > 20%, in US accessible areas, 94% of erosions with > 10% bone loss were detected. They concluded that in accessible areas, the US is quite accurate even for the detection of the smallest erosions in only one plane [26]. US was also introduced in the rheumatology community in Turkey in 2006. Çakılılı et al. performed a survey among 108 rheumatologists (80 consultants, 28 fellows) for using of US in their daily practice. Fifty-six percent of the participants stated that they use US in their daily practice. Twenty-four percent of the participants did not have any training in US and 46% of rheumatologists are still in need of further training in US [27]. In our country, US is frequently used, especially in diagnosis and follow-up of the rheumatoid arthritis erosions. Aydın et al. included 200 patients with rheumatoid arthritis who had US evaluation either for diagnosis or follow-up. The authors investigated the number of joints scanned and compared according to the indications of US. They found that the most common indication was assessing disease activity (48.5%) followed by diagnosis (45.5%). Wrists (66%) and MCPs (63.5) were the most commonly scanned joints followed by knees (26%), PIPs (20%). The authors mentioned that the number of joints assessed by US was significantly higher when used for diagnostic aims as compared to evaluating disease activity and injections guidance [28].

Joint manifestations are common in patients with BD, but destructive arthritis can rarely be seen. If osteo-articular symptoms are predominant, patients should be closely followed-up, and necessary radiological evaluations, especially a US examination should be regularly undertaken. It is considered that US is a convenient diagnostic method for erosive arthritis in patients with BD due to its safe nature, fast results, and cost-effectiveness.

## Data Availability

The datasets generated and/or analysed during the current study are not publicly available due to the patients’ privacy but are available from the corresponding author on reasonable request.

## Abbreviations

BD: Behçet’s disease
US: Ultrasound
MCP: Metacarpophalangeal
MRI: Magnetic resonance imaging
ISG: International study group
